# Nutrigenetic Interactions Might Modulate the Antioxidant and Anti-Inflammatory Status in Mastiha-Supplemented Patients With NAFLD

**DOI:** 10.3389/fimmu.2021.683028

**Published:** 2021-05-07

**Authors:** Stavroula Kanoni, Satish Kumar, Charalampia Amerikanou, Mary Jo Kurth, Maria G. Stathopoulou, Stephane Bourgeois, Christine Masson, Aimo Kannt, Lucia Cesarini, Maria-Spyridoula Kontoe, Maja Milanović, Francisco J. Roig, Mirjana Beribaka, Jonica Campolo, Nuria Jiménez-Hernández, Nataša Milošević, Carlos Llorens, Ilias Smyrnioudis, M. Pilar Francino, Nataša Milić, Andriana C. Kaliora, Maria Giovanna Trivella, Mark W. Ruddock, Milica Medić-Stojanoska, Amalia Gastaldelli, John Lamont, Panos Deloukas, George V. Dedoussis, Sophie Visvikis-Siest

**Affiliations:** ^1^ William Harvey Research Institute, Barts and The London School of Medicine and Dentistry, Queen Mary University of London, London, United Kingdom; ^2^ EA_1122, IGE-PCV, Université de Loraine, Nancy, France; ^3^ Department of Nutrition and Dietetics, School of Health Science and Education, Harokopio University, Athens, Greece; ^4^ Randox Laboratories Ltd (RANDOX), Crumlin, United Kingdom; ^5^ Fraunhofer Institute of Translational Medicine and Pharmacology, Frankfurt, Germany; ^6^ ASST Grande Ospedale Metropolitano Niguarda, Milan, Italy; ^7^ Faculty of Medicine, University of Novi Sad, Novi Sad, Serbia; ^8^ Biotechvana, Parc Científic, Universitat de València, Valencia, Spain; ^9^ Facultad de Ciencias de la Salud, Universidad San Jorge, Zaragoza, Spain; ^10^ Department of Biology, Faculty of Technology Zvornik, University of East Sarajevo, Zvornik, Bosnia and Herzegovina; ^11^ Institute of Clinical Physiology National Research Council, Pisa, Italy; ^12^ Area de Genòmica i Salut, Fundació per al Foment de la Investigació Sanitária i Biomèdica de la Comunitat Valenciana (FISABIO-Salut Pública), Valencia, Spain; ^13^ CIBER en Epidemiología y Salud Pública, Madrid, Spain; ^14^ Chios Mastic Gum Growers Association, Chios, Greece; ^15^ Clinic for Endocrinology, Diabetes and Metabolic Diseases, Clinical Centre of Vojvodina, Novi Sad, Serbia; ^16^ Centre for Genomic Health, Life Sciences, Queen Mary University of London, London, United Kingdom

**Keywords:** non-alcoholic fatty liver disease, inflammation, oxidative stress, Mastiha, nutrigenetics, randomized clinical trial, MAST4HEALTH

## Abstract

Non-alcoholic fatty liver disease (NAFLD) is the most common liver disease with no therapeutic consensus. Oxidation and inflammation are hallmarks in the progression of this complex disease, which also involves interactions between the genetic background and the environment. Mastiha is a natural nutritional supplement known to possess antioxidant and anti-inflammatory properties. This study investigated how a 6-month Mastiha supplementation (2.1 g/day) could impact the antioxidant and inflammatory status of patients with NAFLD, and whether genetic variants significantly mediate these effects. We recruited 98 patients with obesity (BMI ≥ 30 kg/m^2^) and NAFLD and randomly allocated them to either the Mastiha or the placebo group for 6 months. The anti-oxidative and inflammatory status was assessed at baseline and post-treatment. Genome-wide genetic data was also obtained from all participants, to investigate gene-by-Mastiha interactions. NAFLD patients with severe obesity (BMI > 35kg/m^2^) taking the Mastiha had significantly higher total antioxidant status (TAS) compared to the corresponding placebo group (P value=0.008). We did not observe any other significant change in the investigated biomarkers as a result of Mastiha supplementation alone. We identified several novel gene-by-Mastiha interaction associations with levels of cytokines and antioxidant biomarkers. Some of the identified genetic loci are implicated in the pathological pathways of NAFLD, including the lanosterol synthase gene (*LSS)* associated with glutathione peroxidase activity (Gpx) levels, the mitochondrial pyruvate carrier-1 gene (*MPC1)* and the sphingolipid transporter-1 gene (*SPNS1*) associated with hemoglobin levels, the transforming growth factor‐beta‐induced gene (*TGFBI*) and the micro-RNA 129-1 (*MIR129-1)* associated with IL-6 and the granzyme B gene (*GZMB*) associated with IL-10 levels. Within the MAST4HEALTH randomized clinical trial (NCT03135873, www.clinicaltrials.gov) Mastiha supplementation improved the TAS levels among NAFLD patients with severe obesity. We identified several novel genome-wide significant nutrigenetic interactions, influencing the antioxidant and inflammatory status in NAFLD.

**Clinical Trial Registration:**

ClinicalTrials.gov, identifier NCT03135873.

## Introduction

NAFLD is one of the major causes of liver dysfunction worldwide and the most common liver disease in Western populations, characterized by hepatic fat accumulation of more than 5% (1, 2). The condition encompasses a wide spectrum of disorders, starting from mild hepatic fat deposition to more progressive steatosis, accompanied by fibrosis and cirrhosis and progressively resulting in hepatocellular carcinoma (1, 2).

The pathogenesis of NAFLD is not fully elucidated but according to the “multiple parallel hits hypothesis” model, hepatic accumulation of triglycerides triggers multiple conditions in the liver. These contribute synergistically to disease onset and progression, including increased hepatic oxidative stress linked to progressive cell death (3). NAFLD is associated with insulin resistance, type 2 diabetes, obesity and the metabolic syndrome (4). There is also growing evidence that inflammatory markers and oxidative stress play a pivotal role in the pathophysiology of the disease, highlighting their role as potential therapeutic targets (3, 5–7).

Currently, there is no tailored drug treatment for NAFLD. Adopting a healthy lifestyle has been proposed as the cornerstone for disease management (8). Furthermore, dietary interventions aiming to improve the inflammatory profile have proven beneficial in patients with NAFLD (9). There is also growing scientific interest in a variety of supplements and herbal products, as potential therapeutic and/or preventive approaches for NAFLD (10, 11). Mastiha is a natural supplement based on the dried resinous exudate from stems and branches of the tree *Pistacia lentiscus*, exclusively cultivated on Chios Island, Greece. Mastiha contains a plethora of bioactive compounds including phytosterols, terpenes, phenolic compounds, arabino-galactane proteins and a 30% of poly- β-myrcene (12, 13). In vitro and *in vivo* studies have highlighted the antioxidant and anti-inflammatory properties of Mastiha (13) and have indicated a reduction in hepatic steatosis and liver injury in NASH (14).

An individual’s predisposition to NAFLD is affected by both genetic and environmental factors (15). Some 11 genetic loci have been identified to date for their association with NAFLD but explain only 10-20% of the disease’s heritability (16–19). This has led to the hypothesis that gene-by-nutrient interactions might modulate the susceptibility to NAFLD. There is growing evidence from nutrigenomic studies, suggesting that personalized nutrition may represent a customized therapeutic approach to the disease, also incorporating the genetic susceptibility (15, 20). The nutrigenetic research has mainly focused on NAFLD-associated loci, such as PNPLA3, TM6SF2 and GCKR (15, 20). Investigators have also focused on antioxidant defense system genes (21), and the role of miRNAs (22).

MAST4HEALTH is an EU-funded project, investigating the effectiveness of the Mastiha supplement in patients with NAFLD (23). In the current study we assessed: i) the impact of a 6-month Mastiha supplementation on the inflammation and antioxidant profile, and ii) how genetic variants might modulate this effect.

## Materials and Methods

### Study Population

Recruitment took place from 2017 to 2019 in three centers (Department of Dietetics and Nutritional Science, Harokopio University, Athens, Greece, Consiglio Nazionale delle Ricerche Institute of Clinical Physiology, Milano section at Niguarda Hospital, Italy and Faculty of Medicine, University of Novi Sad, Serbia). The participants were men and women, aged 18-67 years, with body mass index (BMI) ≥ 30 kg/m^2^ and with established NAFLD/NASH, based on the sensitive LiverMultiScan magnetic resonance imaging (MRI) technique (24). Exclusion criteria included hepatotoxic medication, concomitant liver disease, decompensated diabetes mellitus [diabetes mellitus type 1, uncontrolled diabetes mellitus type 2 (HbA1c ≥ 7,5%)], thyroid disease, hypopituitarism, Cushing’s syndrome, alcohol abuse [>20 g/day for women and >30 g/day for men), EASL Guidelines (8)] or drug addiction, systemic diseases, pregnancy, lactation, vegan or lacto- and ovo-lacto- vegetarianism, psychiatric or mental disorder, recent loss in body weight or current diet, any use of antioxidant-phytochemical rich supplement, pre- or pro-biotics, changes in drug treatment, antibiotic treatment during or prior to screening. Ethics Committees approvals were obtained from all centers and the trial was conducted according to the rules of the Declaration of Helsinki, and the Data Protection Act 1998. All participants gave written informed consent before inclusion in the study. The full protocol of the trial can be accessed *via* ClinicalTrials.gov (Identifier: NCT03135873).

### Mastiha Supplementation

Participants were randomly allocated to either the Mastiha or the placebo group. The randomization algorithm was designed to balance the size of each group per country and per sex, by picking a pseudo-random number from 0 to 1. All participants and researchers were blinded to the treatment allocation. Mastiha (100% natural) or matching placebo (corn starch) capsules weighing 0.35 g each were given in 3 equal doses daily (total of 2.1 g). No side effect or any discomfort was reported by the participants, while self-reported compliance was monitored biweekly.

### Sample Collection, Anthropometric Measurements, and Medical and Lifestyle Assessment

Blood samples were collected after an overnight fast. Serum was isolated for biomarker quantification. DNA was extracted from 300 μl buffy coat using iPrep™ PureLink^®^ gDNA Blood Kit and iPrep™ Purification Instrument (Invitrogen, Thermo Fischer Scientific). Detailed medical history was recorded including personal/family medical history and medication. Body weight and height were measured to the nearest 0.1 kg and millimeter, respectively and BMI was computed as weight (kg)/height^2^ (m^2^). Physical activity level was evaluated *via* the International Physical Activity Questionnaire (IPAQ) and the IPAQ scoring protocol was applied to estimate Metabolic Equivalent Task minutes/week (MET-min/wk) (25). Participants were asked about their smoking habits and were categorized as current, former or never smokers. All procedures were repeated at baseline and at the end of the trial, except for the DNA isolation that was only performed at baseline.

### Biomarker Measurements

Hemoglobin (ηβ) levels were measured on whole blood samples using a standard hematology analyzer. Serum concentration of total antioxidant status (TAS) (mmol/L) was determined by the chromogenic method by Randox TAS kits (Randox Laboratories Ltd, Crumlin, UK) at Randox Clinical Laboratory Services (Antrim, UK). The RANSOD kit (Randox Laboratories Ltd, Crumlin, UK), was used to quantify SuperOxide Dismutase (SOD) activity, in erythrocyte pellet, on a Randox RX Series Analyser (Randox Laboratories Ltd, Crumlin, UK). SOD activity was expressed as units/gram of hemoglobin (U/g HB). Glutathione peroxidase (Gpx) activity in whole blood samples was determined spectrophotometrically with an automated biochemical analyzer RX-Daytona (Randox Laboratories Ltd, Crumlin, UK) using the Ransel kit (Randox Laboratories Ltd, Crumlin, UK). Serum samples were run on the Randox high sensitivity cytokine I multiplex array (Randox Laboratories Ltd, Crumlin, UK), using an Evidence Investigator analyzer (Randox Laboratories Ltd, Crumlin, UK) according to manufacturer’s instructions. High sensitivity cytokine I multiplex array measures the levels of IL-1α, IL-1β, IL-2, IL-4, IL-6, IL-8, IL-10, MCP-1, TNF-α, INF-γ, EGF and VEGF-A. All measurements were performed at baseline and at the end of the clinical trial.

### Gene Expression

Whole blood collected directly into PAXgene Blood RNA Tubes was used for the following gene expression quantification: B2M, IL-6, IL-1α, TNF-α and VEGF-A (isoforms 121, 165, 189, 145). Reverse transcription was performed using the Iscript cDNA Synthesis (Biorad, France). The cDNA was purified with QIAquick PCR purification Kit (Qiagen France) to generate standards for each gene for absolute quantification and was quantified with nanodrop 1000 (ThermoScientific). Conventional RT-PCR was performed for each gene to generate large quantities of the product which were subsequently purified by running on 10% Polyacrylamide gel as per QIAquik PCR Purification Kit protocol (QIAGEN, Courtaboeuf, France). The purified qPCR products were used to establish the standard ranges for each gene with the NanoDrop 1000 Spectrophotometer (ThermoScientific). Absolute quantification of the genes expression was performed using QuantStudio 3 Applied Biosystems (Life Technologies, France). Gene expression levels were normalized to the B2M housekeeping gene expression. Measurements were performed at baseline and at the end of the clinical trial.

### Genotyping and Imputation

Samples were genotyped on the Illumina GSA microarray at the Queen Mary University of London Genome Centre. Standard quality control (QC) procedures (variants call rate < 98%, Hardy Weinberg Equilibrium (HWE) p-value < 10^-6^) were applied to exclude poorly genotyped variants. The final dataset comprised of 95 samples and 661,221 variants. Principal component analysis was performed on the genotyping dataset and the first 5 principal components (PCs) were retained for use in downstream analysis. Imputation to the HRC (Haplotype Reference Consortium) and 1000 Genome panels was performed on the Michigan server (26). Standard post-imputation QC was applied, all monomorphic and badly imputed variants (INFO<0.4) were removed and the two imputation datasets were merged. Genetic variants that were present in both sets were retained from the set with the higher imputation quality. The merged imputation dataset consisted of 10,307,610 variants.

### Statistical Analysis

We considered the concentrations of 16 circulating biomarkers and 7 gene expression levels in our analysis. All outcomes were natural log transformed and scaled (mean =0, SD=1) prior to statistical analysis. To assess the effect of the Mastiha treatment on each of these biomarkers, we compared the post-treatment mean levels between the Mastiha and the placebo groups, *via* analysis of covariance (ANCOVA) models. The models were adjusted for the corresponding baseline levels of the tested outcome, age, sex and the center of recruitment. Furthermore, we performed a number of sensitivity analyses with a sequential adjustment for the baseline BMI levels, the baseline physical activity levels, the smoking status or the use of antilipidemic, antihypertensive and/or antidiabetic medication. We additionally stratified our samples into two categories based on their baseline BMI levels: Class I obesity (BMI≤ 35 kg/m^2^, n=65) and Class II or III obesity (BMI> 35 kg/m^2^, n=33), as we have previously done (23). All ANCOVA models were implemented in R; adjusted means of selected outcomes were calculated *via* the “lsmeans” package in R. Due to the high correlation among the investigated outcomes, we only considered multiple testing for the 3 model analysis and therefore report significance at P value ≤ 0.016.

For the gene-by-Mastiha interaction analysis, we first applied a linear regression model per outcome and adjusted for the corresponding baseline levels of each biomarker, age, sex, center and the first 5 genetic principal components. We obtained the residuals from each of these linear regression models and scaled them (mean=0, SD=1), before using them as the dependent variable in the gene-by-Mastiha interaction analysis. For each outcome residuals, we performed a genome-wide interaction scan with the GxEScan software (27). Each genetic variant was tested for interaction with the trial group (coded as 1 for the Mastiha group and 0 for the placebo) using the equation: Y= β_0_ + β_G_G + β_E_E + β_GxE_GxE + ϵ. We tested the null hypothesis β_GxE_=0. We excluded interaction association results with <30 samples, a minor allele count <10 and implausible interaction effect sizes (β_GxE_GxE ≥ 4). We report significant interactions at a genome-wide significance level (P value ≤ 5e-08). The index variant per locus was defined at a 500kb window and was annotated with the nearest gene using BioMart from ENSEMBL.

Based on power calculations, we had 80% statistical power to detect a minimum effect size difference (effect size=difference in means/pooled SD), between the two treatment groups at the end of the trial, of 0.55 with a maximum of 52 samples per group. For the interaction analysis, we had 80% power to detect a minimum interaction effect size of 0.7 for a minimum allele frequency of 0.1, among the 87 individuals that completed the trial. Power calculations are illustrated in **Supplementary Figure 1** and were implemented in R and QUANTO, respectively.

## Results

### Baseline Characteristics

Ninety-eight patients were randomized to take Mastiha (n=41) or placebo (n=57) for 6 months (CONSORT FLOW DIAGRAM in **Data Sheet 1**). Out of the 98 volunteers in the MAST4HEALTH trial (NCT03135873) (23), 87 (88.8%) completed the intervention. Baseline characteristics are presented in **Table 1**. We observed nominally significant differences between the two groups at baseline for circulating IL-1α, IL-2, IL-4, and TNF-α (**Table 1**).

**Table 1:** Baseline characteristics of all participants and per trial group.

Baseline Characteristics	All		placebo		Mastiha		
Demographic	n	Mean (SD) or n	n	Mean (SD) or n	n	Mean (SD) or n	P value*
**Age**	98	48.83 (9.36)	57	48.95 (9.04)	41	48.66 (9.89)	0.929
**Gender (M/F)**	98	68/30	57	42/15	41	26/15	0.386
Centre (GR/IT/SR)**	98	38/30/30	57	23/17/17	41	15/13/13	0.931
**Physical Activity**	91	3622.17 (5128.18)	52	3536.78 (5345.85)	39	3736.04 (4889.48)	0.921
**BMI (kg/m^2^)**	98	34.44 (4.41)	57	34.66 (5.05)	41	34.14 (3.38)	0.513
**Smoking (Never/Ex/Current)**	97	51/25/21	56	28/14/14	41	23/11/7	0.642
**Medication**							
**Antilipidemic (Yes/No)**	98	16/82	57	10/47	41	6/35	0.914
**Antihypertensive (Yes/No)**	98	27/71	57	18/39	41	9/32	0.410
**Antidiabetic (Yes/No)**	98	14/84	57	8/49	41	6/35	1.000
**Circulating Markers**							
**EGF (pg/ml)**	96	66.536 (54.811)	56	66.714 (53.183)	40	66.288 (57.698)	0.286
**Gpx (U/L)**	96	8581.101 (3927.79)	55	9079.024 (4303.28)	41	7913.157 (3292.809)	0.221
**HB (g/L)**	96	0.146 (0.013)	55	0.146 (0.012)	41	0.147 (0.013)	0.239
**IL-1α (pg/ml)**	95	0.193 (0.185)	55	0.155 (0.143)	40	0.246 (0.223)	0.024
**IL-1β (pg/ml)**	92	0.964 (0.521)	54	0.883 (0.388)	38	1.079 (0.654)	0.128
**IL-2 (pg/ml)**	87	1.97 (1.712)	49	1.636 (1.55)	38	2.402 (1.832)	**0.011**
**IL-4 (pg/ml)**	97	1.624 (0.539)	56	1.531 (0.377)	41	1.75 (0.688)	0.043
**IL-6 (pg/ml)**	97	1.851 (1.324)	56	1.915 (1.4)	41	1.764 (1.224)	0.472
**IL-8 (pg/ml)**	96	9.095 (5.934)	55	8.39 (5.128)	41	10.04 (6.822)	0.092
**IL-10 (pg/ml)**	96	0.771 (0.471)	55	0.732 (0.459)	41	0.825 (0.488)	0.166
**IFNγ (pg/ml)**	93	0.373 (0.425)	53	0.321 (0.322)	40	0.443 (0.528)	0.055
**MCP-1 (pg/ml)**	97	206.088 (80.808)	56	207.929 (71.485)	41	203.573 (92.922)	0.257
**SOD (U/g HB)**	95	1896.219 (442.514)	54	1874.869 (432.226)	41	1924.338 (459.58)	0.840
**TAS (mmol/L)**	96	1.906 (0.207)	55	1.889 (0.17)	41	1.929 (0.249)	0.157
**TNF-α (pg/ml)**	95	2.701 (1.127)	55	2.461 (0.685)	40	3.031 (1.49)	0.027
**VEGF-A (pg/ml)**	97	151.531 (113.965)	56	157.223 (113.504)	41	143.756 (115.541)	0.304
**Gene expression (copy number/B2M copy number)**							
**TNF-α**	97	1.213 (2.715)	56	1.146 (2.183)	41	1.306 (3.335)	0.773
**IL-1α**	97	0.002 (0.003)	56	0.002 (0.004)	41	0.001 (0.002)	0.773
**IL-6**	97	0.001 (0.001)	56	0.001 (0.001)	41	0.001 (0.001)	0.391
**VEGF-A-121**	97	0.057 (0.077)	56	0.062 (0.095)	41	0.051 (0.041)	0.825
**VEGF-A-165**	97	0.354 (0.441)	56	0.369 (0.484)	41	0.333 (0.378)	0.586
**VEGF-A-145**	97	0.001 (0.003)	56	0.001 (0.004)	41	0.001 (0.003)	0.916
**VEGF-A-189**	97	0.001 (0.002)	56	0.001 (0.002)	41	0.001 (0.002)	0.811

*P value for the trial groups comparison. Outcome comparisons are adjusted for age, sex and center. Differences in categorical variables were assessed with Chi-square test, **GR, Greece; IT, Italy; SR, Serbia. Bold values denote significant results at the multiple testing threshold.

### Effects of the Mastiha Supplementation

**Figure 1:**
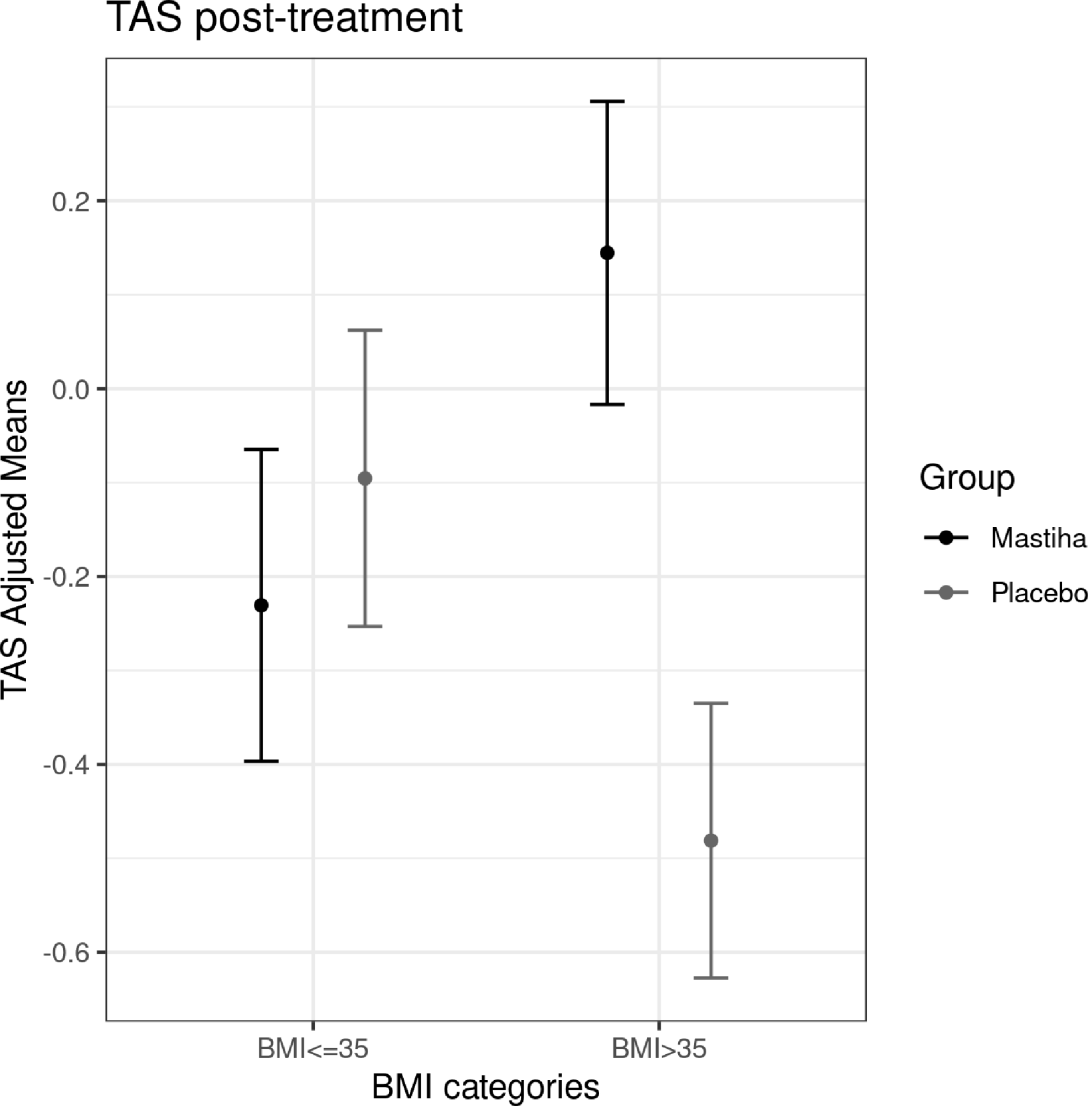
Differences in adjusted means for post-treatment TAS, between the Mastiha and placebo groups, stratified by BMI category. Adjustments were performed for baseline levels of TAS, age, sex and center.

When comparing the post-treatment biomarkers between the two groups we did not identify any significant difference, apart from a trend for higher MCP-1 levels in the Mastiha group (**Supplementary Table 1**). The results remained robust across all different sensitivity analyses, apart from MCP-1 where the trend was attenuated (P value = 0.071) when adjusting our model for the use of medication (antilipidemic, antihypertensive and/or antidiabetic) (**Supplementary Table 1**). Moreover, as in our previous analyses (23), we stratified our individuals by BMI category and the baseline characteristics per trial group are presented in **Table 2**, while the baseline characteristics for all individuals stratified only by BMI category are presented in **Supplementary Table 2**. We found a significant increase (beta=0.626, SE=0.213, P value=0.008) in TAS within the Mastiha group post-treatment compared to the placebo group (mean ± SD: 2.011 ± 0.183; 1.824 ± 0.160 mmol/L, for the Mastiha and placebo groups respectively), albeit only in the BMI>35kg/m^2^ group (n=28) (**Supplementary Table 3** and **Figure 1**). In the BMI ≤ 35kg/m^2^ group (n=59) we observed a decreasing trend in the Gpx levels and an increasing trend for the MCP-1 levels within the Mastiha group (**Supplementary Table 3**). Both became non-significant when we additionally adjusted our models for the use of antilipidemic, antihypertensive and/or antidiabetic medication (beta=-0.340, SE=0.169, P value=0.050 and beta=0.344, SE=0.169, P value=0.047, for Gpx and MCP-1 respectively). The corresponding association for the TAS levels in the BMI>35kg/m^2^ group, after the additional adjustments for medication use, remained significant albeit slightly attenuated (beta=0.602, SE=0.237, P value=0.019).

**Table 2:** Baseline characteristics of all participants stratified by BMI category and trial group.

Baseline Characteristics	placebo (BMI ≤ 35kg/m^2^)		Mastiha (BMI ≤ 35kg/m^2^)			placebo (BMI>35kg/m^2^)		Mastiha (BMI>35kg/m^2^)		
Demographic	n	Mean (SD)	n	Mean (SD)	P value*	n	Mean (SD)	n	Mean (SD)	P value*
**Age**	37	49.08 (9.08)	28	47.89 (10.09)	0.563	20	48.7 (9.21)	13	50.31 (9.6)	0.626
**Gender (M/F)**	37	29/8	28	18/10	0.328	20	13/7	13	8/5	1.000
Centre (GR/IT/SR)**	37	9/16/12	28	8/11/9	0.918	20	14/1/5	13	7/2/4	0.508
**Medication**										
**Antilipidemic (Yes/No)**	37	8/29	28	3/25	0.408	20	2/18	13	3/10	0.598
**Antihypertensive (Yes/No)**	37	12/25	28	6/22	0.483	20	6/14	13	3/10	0.971
**Antidiabetic (Yes/No)**	37	5/32	28	4/24	1.000	20	3/17	13	2/11	1.000
**Circulating Markers**										
**EGF (pg/ml)**	37	79.2432 (55.7292)	28	67.5536 (54.7246)	0.256	19	42.3158 (38.4698)	12	63.3333 (66.6229)	0.789
**Gpx (U/L)**	36	9024.5998 (3310.18)	28	7274.5589 (2577.337)	0.070	19	9182.1424 (5850.7827)	13	9288.5992 (4265.5518)	0.755
**HB (g/L)**	37	0.1476 (0.0113)	28	0.1461 (0.0143)	0.810	18	0.1426 (0.014)	13	0.1481 (0.0107)	0.079
**IL-1α (pg/ml)**	37	0.1478 (0.15)	27	0.2181 (0.1816)	0.050	18	0.1689 (0.1293)	13	0.3027 (0.2904)	0.374
**IL-1β (pg/ml)**	35	0.9 (0.3941)	27	1.0502 (0.5898)	0.212	19	0.8505 (0.3852)	11	1.15 (0.8192)	0.328
**IL-2 (pg/ml)**	33	1.8261 (1.7162)	27	2.572 (1.9241)	0.043	16	1.2431 (1.077)	11	1.9841 (1.5884)	0.097
**IL-4 (pg/ml)**	37	1.5468 (0.4372)	28	1.6768 (0.5146)	0.200	19	1.5005 (0.2237)	13	1.9085 (0.9708)	0.095
**IL-6 (pg/ml)**	37	1.8114 (1.6047)	28	1.8014 (1.3404)	0.717	19	2.1168 (0.8773)	13	1.6846 (0.9711)	0.065
**IL-8 (pg/ml)**	36	8.296 (5.2103)	28	10.0396 (6.1648)	0.043	19	8.5684 (5.1039)	13	10.0392 (8.3443)	0.658
**IL-10 (pg/ml)**	36	0.7789 (0.5153)	28	0.8529 (0.5039)	0.203	19	0.6421 (0.3191)	13	0.7646 (0.4638)	0.598
**IFNγ (pg/ml)**	36	0.3167 (0.2156)	27	0.4706 (0.6147)	0.158	17	0.3294 (0.4853)	13	0.3846 (0.2881)	0.268
**MCP-1 (pg/ml)**	37	207.9189 (77.0438)	28	212.8571 (76.8753)	0.631	19	207.9474 (61.1773)	13	183.5769 (121.8609)	0.091
**SOD (U/g HB)**	36	1938.8874 (399.9199)	28	1894.607 (423.6875)	0.397	18	1746.8333 (476.5706)	13	1988.3751 (541.8856)	0.383
**TAS (mmol/L)**	36	1.9026 (0.1783)	28	1.9414 (0.2612)	0.071	19	1.8629 (0.1557)	13	1.9031 (0.2275)	0.948
**TNF-α (pg/ml)**	36	2.5357 (0.7288)	27	3.1887 (1.5812)	0.061	19	2.3195 (0.5845)	13	2.7019 (1.2732)	0.210
**VEGF-A (pg/ml)**	37	167.2568 (129.1862)	28	154.6071 (128.9989)	0.570	19	137.6842 (73.3281)	13	120.3846 (78.6827)	0.430
**Gene expression (copy number/B2M copy number)**										
**TNF-α**	37	1.1638 (2.5612)	28	1.6494 (3.9887)	0.718	19	1.1111 (1.2022)	13	0.5651 (0.6318)	0.236
**IL-1α**	37	0.0023 (0.0047)	28	0.0015 (0.0019)	0.506	19	0.0011 (0.0016)	13	0.0012 (0.0011)	0.197
**IL-6**	37	0.0011 (0.0012)	28	0.0012 (0.0015)	0.960	19	0.0012 (0.0008)	13	0.0008 (0.0007)	0.149
**VEGF-A-121**	37	0.0735 (0.1149)	28	0.053 (0.0451)	0.631	19	0.0395 (0.0229)	13	0.0477 (0.0316)	0.691
**VEGF-A-165**	37	0.4229 (0.5693)	28	0.3504 (0.4335)	0.697	19	0.2639 (0.2262)	13	0.2939 (0.2255)	0.795
**VEGF-A-145**	37	0.0016 (0.0044)	28	0.0018 (0.0035)	0.964	19	0.001 (0.0014)	13	0.0006 (0.001)	0.854
**VEGF-A-189**	37	0.0016 (0.0025)	28	0.0015 (0.0021)	0.943	19	0.0011 (0.001)	13	0.0012 (0.0009)	0.565

*P value for the trial groups comparison. Outcome comparisons are adjusted for age, sex and center. Differences in categorical variables were assessed with Chi-square test, **GR, Greece; IT, Italy; SR, Serbia.

### Genetic Interaction Association Effects of Mastiha

**Figure 2:**
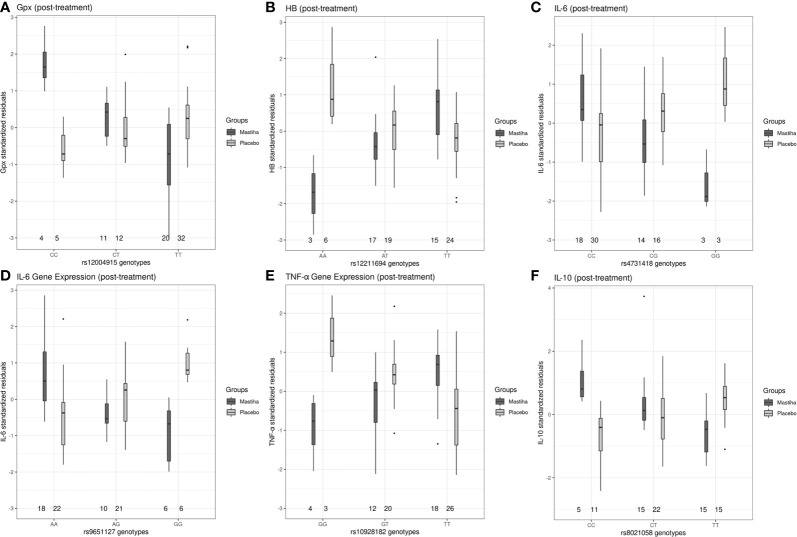
Boxplots of selected post-treatment levels (adjusted for the baseline levels, age, sex, center and the first 5 genetic principal components) between the Mastiha and placebo groups, stratified by genotype, for the significant gene-by-Mastiha interactions (outliers are presented as dots: **(A)** Gpx levels by the rs12004915 genotypes, **(B)** HB by rs12211694, **(C)** IL-6 by rs4731418, **(D)** IL-6 by rs9651127, **(E)** TNF-α by rs10928182 and **(F)** IL-10 by rs8021058.

When we investigated how genetic variants might modulate the effect of Mastiha on biomarkers of inflammation and antioxidant status, we detected several interaction associations reaching genome-wide significance (**Table 3**). Regional plots for all significant interactions are presented in **Supplementary Figure 2**. For the post-treatment Gpx activity, we found 2 genetic variants (rs12004915 in the MLLT3 locus and rs2001809 in the LSS locus) with significant interactions in relation to the Mastiha treatment (**Table 3** and **Supplementary Figures 2A, B**). Carriers of the major allele (C) for the MLLT3 locus variant within the Mastiha group had a tendency for higher post-treatment Gpx activity levels compared to homozygous for the T allele from both groups (**Figure 2A**). Similarly, carriers of the major allele (T) for the LSS locus variant had a trend for lower post-treatment Gpx levels (**Supplementary Figure 3A**). When we investigated potential gene-by-Mastiha interaction associations with plasma HB levels, we identified two genetic variants (rs12211694 and rs1548454) at genome-wide significance level (**Table 3** and **Supplementary Figures 2C, D**). For both these variants the major allele showed a tendency for lower post-treatment HB levels among the Mastiha group compared to those not carrying this allele from both groups (**Figure 2B** and **Supplementary Figure 3B**). We also observed that carriers of the A allele of the rs1878686 variant in the SPOCK3 locus (**Supplementary Figure 2E**) had a trend for lower post-treatment TAS levels in the Mastiha group compared to those not carrying this allele from both treatment groups (**Table 3** and **Supplementary Figure 3C**).

**Table 3:** Significant genetic variants-by-Mastiha group interaction results for selected antioxidant and inflammatory biomarkers (per SD).

Outcome	Variant	Coordinates^*^	Reference/Other Allele	n	Interaction beta	Interaction SE	Interaction P value	RAF^**^	Info^$^	Nearest Gene
**Gpx**	rs12004915	9:20274536	C/T	84	1.602	0.264	**1.39E-09**	0.77	1.00	*MLLT3*
**Gpx**	rs2001809	21:47631199	T/C	84	-1.849	0.322	**9.14E-09**	0.72	0.85	*LSS*
**HB**	rs12211694	6:166671763	A/T	84	-1.662	0.291	**1.07E-08**	0.69	0.91	*GNG5P1*
**HB**	rs1548454	16:28043772	G/A	84	-1.724	0.313	**3.55E-08**	0.61	0.83	*GSG1L*
**TAS**	rs1878686	4:168472370	A/C	83	-1.530	0.268	**1.15E-08**	0.56	0.86	*SPOCK3*
**IL-6**	rs13173271	5:135575294	G/A	84	-1.556	0.270	**7.84E-09**	0.60	0.99	*TRPC7*
**IL-6**	rs4731418	7:127845936	G/C	84	-1.676	0.305	**3.79E-08**	0.73	0.88	*MIR129-1/LEP*
**IL-6 Gene expression**	rs9651127	1:230754222	G/A	83	-1.495	0.263	**1.40E-08**	0.65	0.95	*COG2*
**TNF-α Gene expression**	rs10928182	2:144270623	G/T	83	-1.711	0.287	**2.52E-09**	0.74	0.98	*ARHGAP15*
**TNF-α Gene expression**	rs1560294	11:44522418	C/G	83	-1.501	0.267	**1.89E-08**	0.50	0.98	*CD82*
**IL-10**	rs12173570	6:151957714	T/C	83	2.138	0.370	**7.35E-09**	0.86	1.00	*ESR1*
**IL-10**	rs8021058	14:25109630	C/T	83	1.483	0.270	**3.85E-08**	0.60	1.00	*GZMB*

^*^Chromosome: Position (hg19), ^**^Reference allele frequency, ^$^Imputation quality. Bold values denote significant results at the multiple testing threshold.

Furthermore, carriers of the G allele for the rs13173271 (**Supplementary Figure 3D**) in TRPC7 locus (**Supplementary Figure 2F**) or the rs4731418 (**Figure 2C**) in the MIR129-1/LEP locus (**Supplementary Figure 2G**), within the Mastiha group, had a trend for lower post-treatment IL6 levels compared to homozygous of the alternate allele in both groups (**Table 3**). Similarly, we detected that the G allele of the rs9651127 variant in the COG2 locus (**Table 3** and **Supplementary Figure 2H**) had a tendency for lower post-treatment IL-6 gene expression among those taking the Mastiha treatment compared to those not carrying the G allele from all groups (**Figure 2D**). For the post-treatment levels of TNF-α gene expression we identified two loci, ARHGAP15 and CD82 (**Supplementary Figures 2I, J**), showing significant interaction association with the Mastiha treatment (**Table 3**). In particular, the G allele of the rs10928182 in the ARHGAP15 locus (**Figure 2E**) and the C allele of the rs1560294 variant in the CD82 locus (**Supplementary Figure 3E**) were trending for decreased post-treatment levels of the TNF-α gene expression in the Mastiha group, compared to those homozygous for the alternate alleles. In relation to post-treatment IL-10 levels, we found two genetic variants, rs12173570 in the ESR1/CCDC170 locus and rs8021058 in the GZMH/GZMB, with significant interaction association with the Mastiha treatment (**Table 3** and **Supplementary Figures 2K, L**). In particular, carriers of the T allele of the rs12173570 (**Supplementary Figure 3F**) or the C allele of the rs8021058 (**Figure 2F**) within the Mastiha group, had a trend for higher IL-10 post-treatment levels compared to homozygous of the alternate allele in both treatment groups.

## Discussion

Oxidative stress is a hallmark of NAFLD, inducing cytokine production and inflammatory response from both the innate and adaptive immune system (7). Genetic variation, microRNAs, gut microbiota dysbiosis, dietary and lifestyle factors have also been implicated in the pathology of NAFLD (7, 15). Among the potential non-pharmaceutical treatments for NAFLD, there is evidence that Mastiha could be beneficial, due to its antioxidant and anti-inflammatory effects (13). The MAST4HEALTH consortium undertook a multicenter randomized double-blind placebo-controlled clinical trial to investigate the effectiveness of Mastiha supplement as a non-pharmaceutical intervention for managing NAFLD (23). Here we report the effect of Mastiha on the antioxidant and inflammatory status, also considering genetic interactions. To our knowledge, this is the first study to investigate gene-by-Mastiha interactions, with a genome-wide approach, in a clinical trial design.

We assessed the effect of Mastiha on overall antioxidant and inflammation biomarkers in MAST4HEALTH and we detected a significant improvement in TAS, only among the severely obese patients. TAS is considered an accurate indicator of the anti-oxidative status and it has been reported in the literature that in obesity with NAFLD, there is a decline in TAS (28). Our results suggest a beneficial effect of the Mastiha on the overall antioxidant status in NAFLD with severe obesity. We also observed a trend for higher MCP-1 levels among the NAFLD patients receiving the Mastiha, however this trend disappeared when we took into consideration the type of medication that our volunteers were taking. Previous studies with Mastiha have failed to show a significant effect on MCP-1 levels (13). On the other hand, MCP-1 is known to be increased in patients with non-alcoholic fatty liver and is implicated in the progression to non-alcoholic steatohepatitis (29). Several factors influence the levels of MCP-1 (30), including lipid-lowering medication, several antihypertensive and antidiabetic drugs (31, 32). The most important source of oxidative stress in NAFLD is located in the mitochondria and is the result of increasing lipid accumulation. To counteract this, there is evidence of an increased enzyme activity of SOD and Gpx in patients with NAFLD (7). In our study, we identified two significant gene-by-Mastiha interaction associations; in the MLLT3 and LSS loci. *LSS* is of particular interest, as it encodes the lanosterol synthase, which catalyzes the conversion of (S)-2,3 oxidosqualene to lanosterol (the first step in the biosynthesis of cholesterol) and is a member of the terpene cyclase/mutase family (33). Interestingly, cholesterol metabolism in NAFLD is characterized by increased synthesis (34). Oxidative stress has been reported to increase the level of lanosterol in mitochondria, suggesting that this sterol metabolite may be part of a global cellular response to stress (35). We found that the T allele of rs2001809 had a tendency for decreased Gpx activity among participants taking Mastiha. The same allele has also been associated with decreased *LSS* expression (36). Similarly to Mastiha, the methanol leaf extract of *T. indica* was found to regulate the expression of *LSS* (35).

Several studies have shown an association between HB and NAFLD (37, 38). Patients with NAFLD tend to have higher levels of HB compared to controls (38) and HB levels positively correlate with the degree of hepatic steatosis (37). One possible explanation is that HB acts as an antioxidant and is protective in the presence of steatosis (37). We found a significant interaction association at the GNG5P1 locus, where carriers of the A allele for rs12211694 tended to have lower HB among the Mastiha group only. The same allele has been associated with lower expression of the *MPC1* (Mitochondrial Pyruvate Carrier-1) gene in whole blood (36, 39, 40). The *MPC1* gene encodes a protein, part of the MPC1/MPC2 heterodimer, responsible for transporting pyruvate from the cytoplasm into mitochondria (41). While downregulation of the *MPC1* expression seems to be detrimental for several forms of cancer, NASH/NAFLD phenotypes seem to benefit by the MPC disruption (41, 42). This effect is mediated by the decreased amount of pyruvate metabolized to acetyl-CoA and the decreased mitochondrial ROS production (42). Even though we do not have evidence of the mechanism, we speculate that the observed gene-by-Mastiha interaction could be mediated through transcription factors, protein interactions or post-transcriptional modifications (41).

Another significant gene-by-Mastiha interaction for HB levels was detected at the GSG1L locus, where carriers of the G allele for the rs1548454 variant showed a trend for lower post-treatment HB levels in the Mastiha group. The same allele has been previously associated with reduced expression of the *SPNS1* gene (39), which encodes the sphingolipid transporter-1 protein. Sphingolipids, and ceramides as a central module in the sphingolipids metabolism, are also involved in the pathogenesis of NAFLD (43). The pathway includes the link between triglycerides and sphingolipids synthesis, the abnormal hepatic lipid accumulation, the inflammation-induced production of ceramide in the liver and the pro-apoptotic effect of the sphingolipids in the hepatocytes (43). We speculate that Mastiha compounds interact with the protein expression-lowering allele in that locus, resulting in decreased hepatic accumulation of sphingolipids. In both loci for HB, *MPC1* and *SPNS1*, we assume that the resulting decrease in hepatic oxidation and inflammation, would decrease the levels of HB as a consequence.

The role of IL-6 in the pathogenesis of NAFLD has been controversial; some evidence suggests that IL-6 could promote hepatocyte proliferation and have a protective role in liver fibrosis, while other observations point to a positive correlation between IL-6 and NAFLD severity (3, 6). We identified two genetic loci with evidence of a Mastiha nutrigenetic association with circulating IL-6. The major allele of rs13173271 (TRPC7 locus) was found to have a trend for lower IL-6 in the Mastiha group. The same allele has been associated with increased expression of the *TGFBI* gene in the liver (44). The gene encodes the transforming growth factor‐beta‐induced protein, which has been strongly associated with NAFLD and cirrhosis (45). The second locus was in the proximity of MIR129-1/LEP genes. Both genes are very relevant to NAFLD. *MIR129-1* belongs to the microRNAs (miRNAs), short non-protein coding, single-stranded RNAs, that are important for the gene expression regulation (46). Several studies have suggested the potential diagnostic/prognostic value of these biomarkers in NAFLD, as miRNAs seem to be implicated in the progression of the disease (47, 48). Recent studies also highlight the importance of miR-129-5p (transcribed by *MIR129-1*) in improving liver function and suppressing inflammation and liver fibrosis in alcoholic liver disease (49) and NASH animal models (50). On the other hand, the nearby *LEP* gene, encoding leptin, could also be a good biological candidate for our observed nutrigenetic interaction. Leptin is not only a hormone but also a pro-inflammatory cytokine (51). Even though the role of leptin in NAFLD pathogenesis is not fully understood, leptin levels have been recently associated with the NAFLD fibrosis score (52).

IL-10 is an anti-inflammatory cytokine that has a protective role in hepatic steatosis and attenuates hepatocyte damage (6). In our study, we identified a gene-by-Mastiha interaction association for the IL-10 levels in the GZMB locus. Carriers of the C allele for rs8021058 for participants taking Mastiha, had a tendency for higher circulating IL-10 levels. The same allele has been associated with increased expression levels of the *GZMB* gene (40). The gene encodes a member of the granzyme subfamily of proteins, which is secreted by natural killer cells and cytotoxic T lymphocytes. The protein has been implicated in coronary artery disease and cardiac fibrosis (53). Interestingly, in a recent study investigating CD8^+^ T cells in mice under obese/non-obese NASH, gene expression of *GZMB* and *IL-10* was significantly higher in the obese NASH model, suggesting a common pathway of regulation (54).

A major limitation of our study is the relatively small number of individuals enrolled in the clinical trial. This prevented us from undertaking a formal genotype-stratified analysis in the Mastiha and placebo groups. The duration of the trial might not have been long enough to observe significant changes on the oxidative and inflammation status. Furthermore, we only assessed circulating levels of oxidation and inflammation biomarkers, which might have been influenced by other factors and underlying conditions other than NAFLD. Similarly, while we tried to include as many biomarkers as possible in the scope of this clinical trial, the panel was by no means exhaustive.

We showed a potential beneficial antioxidant effect of Mastiha among the severely obese NAFLD patients. We also detected several significant nutrigenetic interactions, relating to both the antioxidant and inflammatory status in NAFLD. The identified loci included genes implicated in the pathophysiology of the disease. Further functional studies are essential to validate our preliminary findings, as potential targets for non-pharmaceutical interventions in NAFLD.

## Data Availability Statement

The datasets presented in this article are not readily available because we are not permitted to make publicly available the genotypic or phenotypic data of the volunteers due to legal and ethical restrictions. The data sets generated and/or analyzed during the current study are available from the MAST4HEALTH Consortium Steering Committee upon request. Requests to access the datasets should be directed to GD, dedousi@hua.gr.

## Ethics Statement

Ethics Committees approvals were obtained from all centers, HUA (Bioethics Committee 49/29-10-2015), CNR (Ethical Clearance by Commissione per l’Etica e l’Integrità nella Ricerca, February 2016), and Niguarda Hospital Ethics Committee 230-052017 (Comitato Etico Milano Area 3-11.05.2017), UNS (Faculty of Medicine Novi Sad, The Human Research Ethics Commission No. 01-39/58/1-27.06.2016). The trial was conducted following the Helsinki Declaration and the Data Protection Act 1998, and was registered with ClinicalTrials.gov (Identifier: NCT03135873). The patients/participants provided their written informed consent to participate in this study.

## Author Contributions

JL, PD, GD, and SV-S conceived and supervised the current analysis. SKa performed the statistical analysis. SKa, SKu, and SV-S wrote the manuscript. SKa and SV-S had primary responsibility for the final content. SKu, CM, MK, SB, and MR performed the measurements and processed data. CA, MK, MS, M-SK, and MR provided substantial revisions to the manuscript. SB, AK, FR, NJ-H, CL, MF, ACK, MT, MM-S, AG, and PD reviewed the manuscript and provided edits. NMilić, ACK, MT, MM-S, AG, and GD supervised the clinical trial. SKu, CA, MK, SB, CM, LC, MM, MB, JC, and NM conducted research. AK and IS provided essential material. All authors contributed to the article and approved the submitted version.

## Funding 

This project received funding from the European Union’s Horizon 2020 research and innovation program MAST4HEALTH under the Marie Skłodowska-Curie grant agreement no 691042. SV-S was supported partly by Agence Nationale de la Recherche, programme d’Investissements d’avenir (PIA), grant number ANR-15RHU-0004 and GEENAGE project (Functional Genomic, Epigenomic and ENvironment interplay to IMPACT management of healthy and pathological AGEing)” within the frames of the French PIA project “Lorraine Université d’Excellence”—LUE, reference ANR-15-IDEX-04-LUE. PD: This work was part of the research themes contributing to the translational research portfolios of the Barts Biomedical Research Centre funded by the UK National Institute for Health Research (NIHR).

## Conflict of Interest

Authors MK, MR, and JL were employed by the company Randox Laboratories Ltd (RANDOX), author AK was employed by the company Fraunhofer Institute of Translational Medicine and Pharmacology, author IS was employed by the company Chios Mastic Gum Growers Association, and authors FR and CL were employed by the company Biotechvana.

The remaining authors declare that the research was conducted in the absence of any commercial or financial relationships that could be construed as a potential conflict of interest.

## Abbreviations

ANCOVA, analysis of covariance; BMI, body mass index; EGF, epidermal growth factor; Gpx, glutathione peroxidase; HB, hemoglobin; HRC, haplotype reference consortium; GSA, global screening array; IFN-γ, interferon-γ; IL, Interleukin; IPAQ, international physical activity questionnaire; MCP-1, monocyte chemoattractant protein-1; MET, metabolic equivalent; MRI, magnetic resonance imaging; NAFLD, non-alcoholic fatty liver disease; NASH, non-alcoholic steatohepatitis; PC, principal component; RT-PCR, reverse transcription polymerase chain reaction, SOD, superoxide dismutase; TAS, total antioxidant status; TNF-α, tumor necrosis factor-α; VEGF-A, vascular endothelial growth factor A.
