# Supplementary figures and images for: Nutrigenetic Interactions Might Modulate the Antioxidant and Anti-Inflammatory Status in Mastiha-Supplemented Patients With NAFLD
Source: Front Immunol. 2021 May 7;12:683028. doi: 10.3389/fimmu.2021.683028 (PMC8138178; doi:10.3389/fimmu.2021.683028)

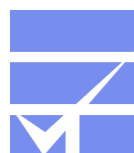

## CONSORT 2010 Flow Diagram

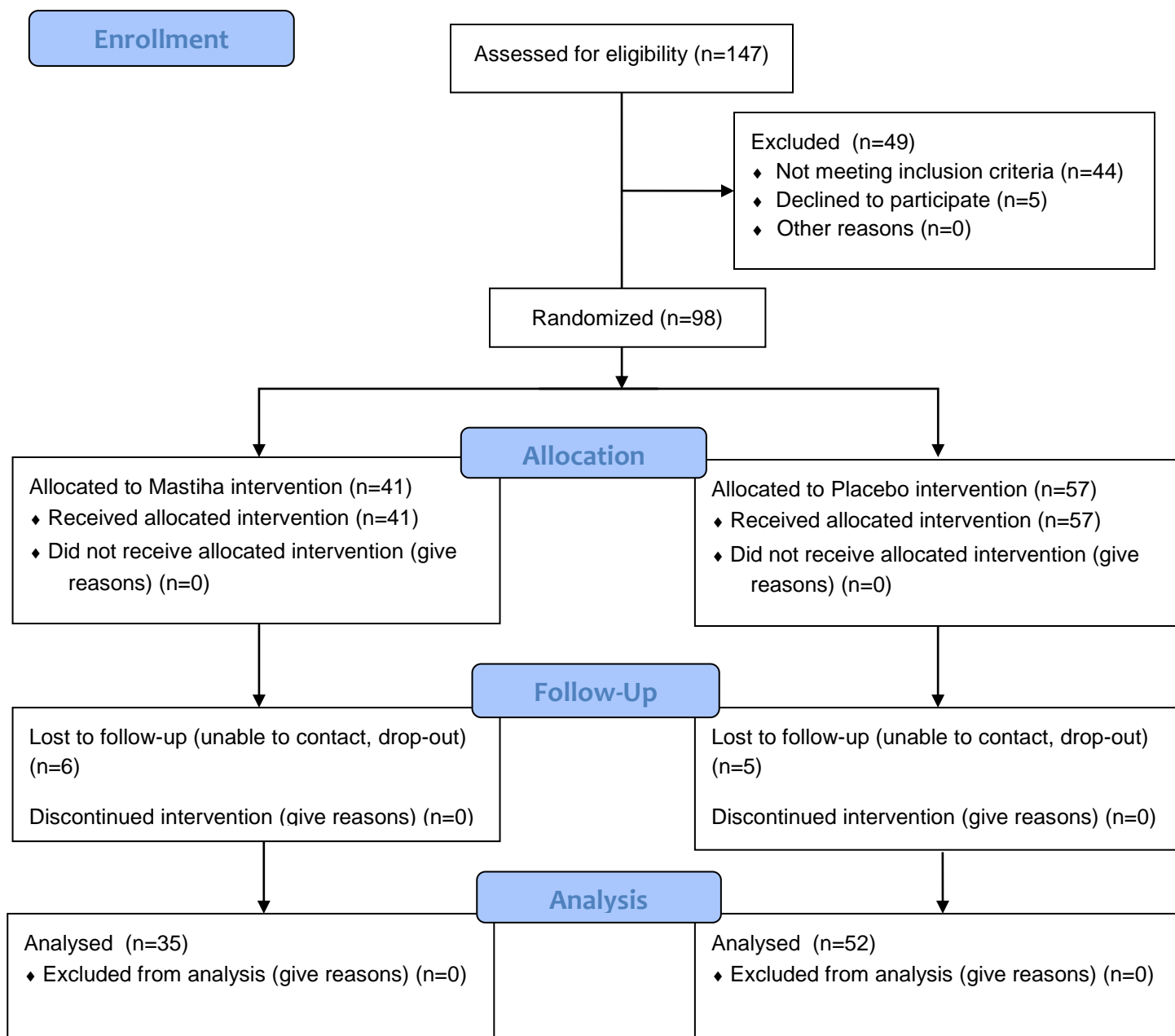

Supplement: Supplementary file 1 [file DataSheet_1.pdf]

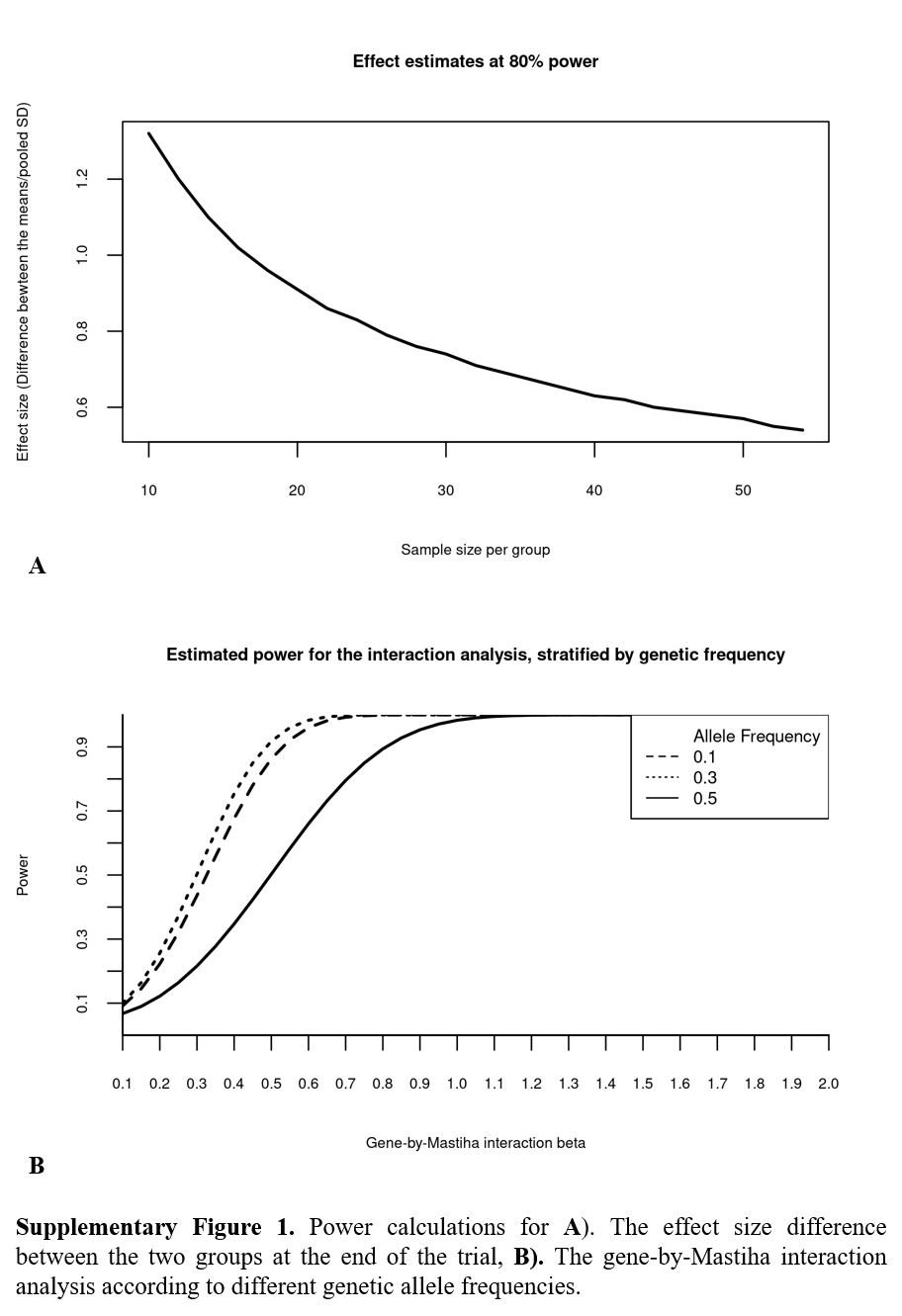

Supplement: Supplementary file 2 [file Image_1.tif]

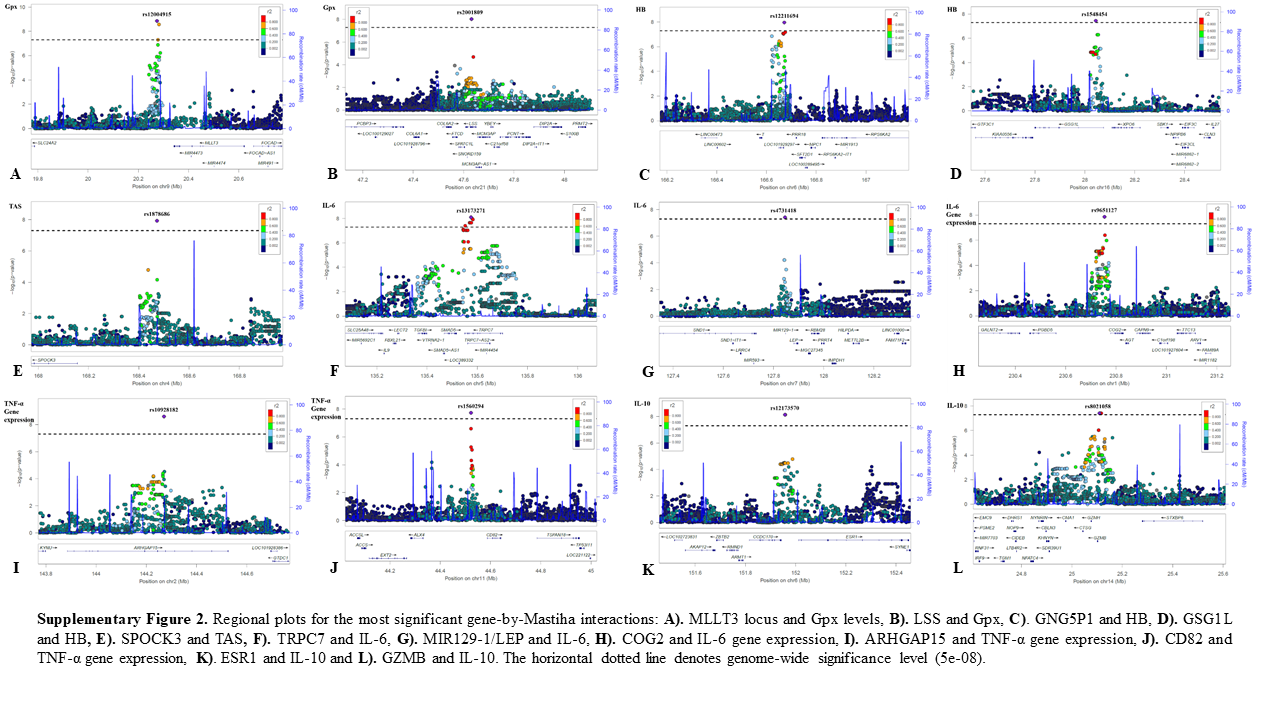

Supplement: Supplementary file 3 [file Image_2.tif]

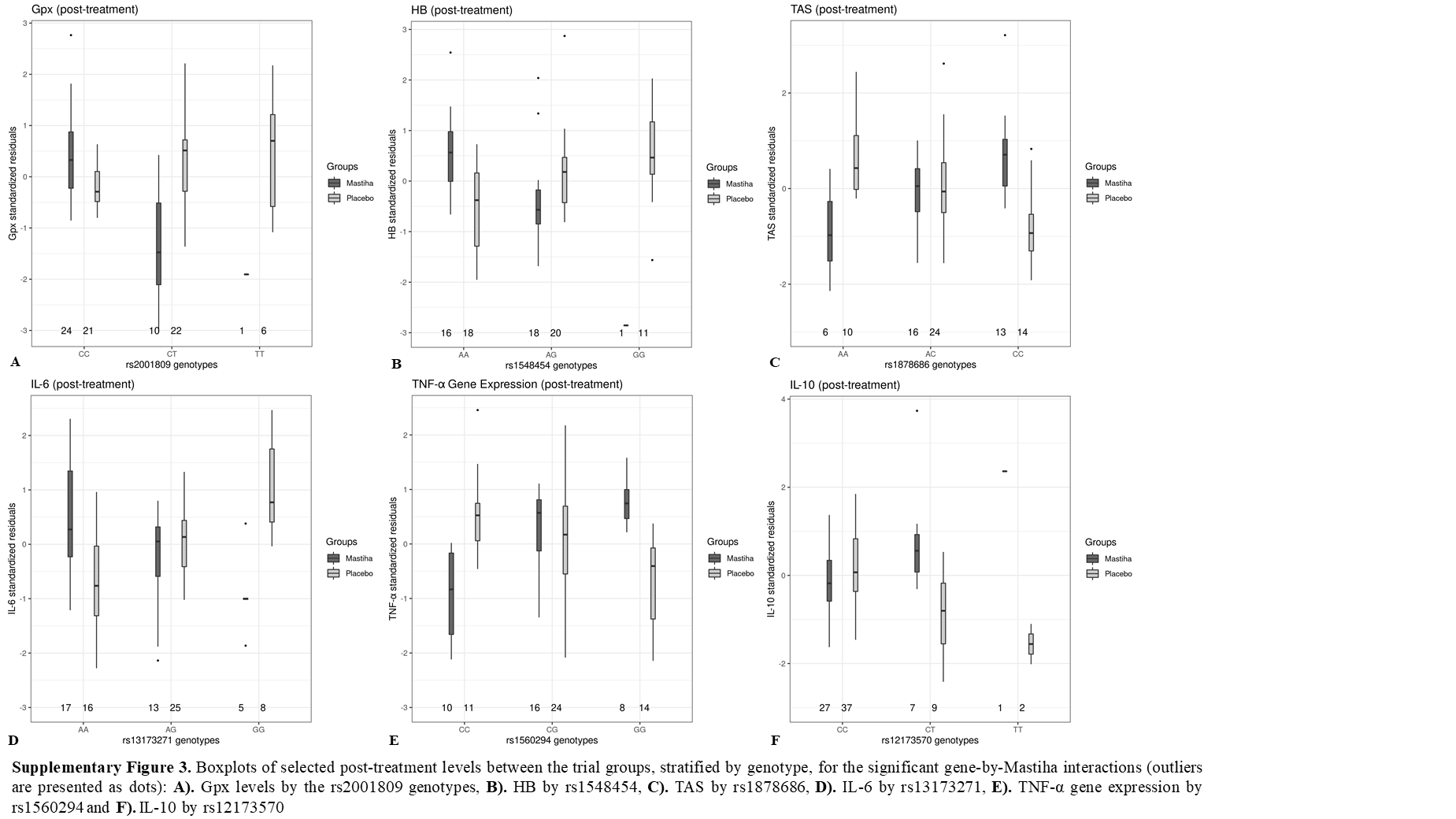

Supplement: Supplementary file 4 [file Image_3.tif]
